# Flexible mate choice when mates are rare and time is short

**DOI:** 10.1002/ece3.666

**Published:** 2013-07-22

**Authors:** Robin M Tinghitella, Emily G Weigel, Megan Head, Janette W Boughman

**Affiliations:** 1 Department of Biological Sciences, University of Denver102 Olin Hall, Denver, Colorado, 80208; 2 Department of Zoology, Michigan State University203 Natural Science Building, East Lansing, Michigan, 48824-1115; 3 Centre for Ecology and Conservation, Biosciences, College of Life and Environmental Sciences, University of ExeterPenryn, Cornwall, TR10 9EZ, U.K; 4 Department of Zoology, Michigan State University203 Natural Science Building, East Lansing, Michigan 48824-1115

**Keywords:** Age, mate choice, operational sex ratio, plasticity, stickleback

## Abstract

Female mate choice is much more dynamic than we once thought. Mating decisions depend on both intrinsic and extrinsic factors, and these two may interact with one another. In this study, we investigate how responses to the social mating environment (extrinsic) change as individuals age (intrinsic). We first conducted a field survey to examine the extent of natural variation in mate availability in a population of threespine sticklebacks. We then manipulated the sex ratio in the laboratory to determine the impact of variation in mate availability on sexual signaling, competition, and mating decisions that are made throughout life. Field surveys revealed within season heterogeneity in mate availability across breeding sites, providing evidence for the variation necessary for the evolution of plastic preferences. In our laboratory study, males from both female-biased and male-biased treatments invested most in sexual signaling late in life, although they competed most early in life. Females became more responsive to courtship over time, and those experiencing female-biased, but not male-biased sex ratios, relaxed their mating decisions late in life. Our results suggest that social experience and age interact to affect sexual signaling and female mating decisions. Flexible behavior could mediate the potentially negative effects of environmental change on population viability, allowing reproductive success even when preferred mates are rare.

## Introduction

The sexual selection literature largely emphasizes exaggerated sexual signals and strong female preferences for those signals (Kirkpatrick and Ryan 1991; Andersson 1994). Yet, in many systems mate choice is extremely variable (Jennions and Petrie 1997). Understanding how and when this variation is expressed is important because mating decisions can influence the rate and direction of evolution by sexual selection (Lande 1981; Kirkpatrick 1982) and ultimately diversification and speciation (Jennions and Petrie 1997; Boughman 2001; Panhuis et al. 2001; Boughman et al. 2005).

While some variation in mate choice may be due to differences in individual preferences and individuals' ability to choose, much may also be due to adaptive phenotypic plasticity, which allows animals to adjust their mating behavior in response to extrinsic and intrinsic cues. An important consequence of this flexibility is the ability to deal with changed or variable environmental circumstances. Faced with changed conditions, animals can disperse, adjust through phenotypic plasticity, or adapt through genetic change. Evolution frequently takes too long to keep up with the pace of ecological change, so plasticity is often the first response (West-Eberhard 2003; Tuomainen and Candolin 2011; Candolin and Wong 2012). Flexible behavior can both increase the probability of surviving and reproducing in changed environments, and provide time for genetic changes to take place (evolutionary rescue; Gomulkiewicz and Holt 1995). For these reasons, it is important to understand how -individuals adjust their mating behavior in response to environmental variation.

Empirical studies across a wide variety of taxa have demonstrated that mate choice can be extremely flexible. Mating decisions depend on intrinsic attributes of the chooser like condition, reproductive state, age, and mating history (e.g., Prosser et al. 1997; Moore and Moore 2001; Hunt et al. 2005; Lynch et al. 2005; Burley and Foster 2006), as well as extrinsic circumstances including predation, ambient light, seasonal changes, and the quality and availability of mates (e.g., Milinski and Bakker 1992; Hedrick and Dill 1993; Forslund and Part 1995; Godin and Briggs 1996; Jirotkul 1999; Kvarnemo and Simmons 1999; Gamble et al. 2003; Borg et al. 2006; Shine et al. 2006; Milner et al. 2010). Recently, both empirical and theoretical work has emphasized understanding how prior experience with male signals alters female mating decisions and the evolution of mate choice in dynamic environments (e.g., Bailey and Zuk 2009; Wong et al. 2011; Bailey and Moore 2012). Studies that investigate how experience interacts with factors intrinsic to the chooser to determine mating decisions, however, are rare. This is unfortunate because plastic responses may change with age, for instance, as the costs and benefits of various mating decisions change (Real 1990; Tuomainen and Candolin 2011). In this study, we ask whether and how mate availability (an extrinsic effect) interacts with age (an intrinsic effect) to determine mate choice decisions. Our measure of mate availability is the operational sex ratio (OSR): the number of receptive females relative to the number of competing males (Emlen and Oring 1977). The OSR can determine both the opportunity for and strength of sexual selection under some circumstances (Emlen and Oring 1977; Kvarnemo and Ahnesjo 1996; Weir et al. 2011; but see Klug et al. 2010). In the simplest case, we might expect mate availability to affect the costs of sampling such that when the chosen sex is rarer, at low density and female-biased OSRs, there are (1) increased distance, energy, time, and predation costs associated with locating mates (Real 1990) and (2) an increased risk of failure to mate by the common sex (Kokko and Mappes 2005). These conditions should lead to reduced choosiness and the evolution of adaptations that allow females to adjust levels of choosiness (adaptive plasticity) when females are the choosier sex.

Importantly, mate availability can change within an individual's lifetime, and over the course of even one breeding season (e.g., Forsgren et al. 2004; Kasumovic et al. 2008). Life-history theory predicts that as individuals approach the end of their reproductive lives, they should be less choosy because fewer opportunities for mating remain (Real 1990). It is reasonable, then, to expect experience with the social mating environment to impact mating decisions of young and old individuals differently. Despite that, variation in experience has only rarely been placed in the context of seasonality or life-history, and when it has, it has been difficult to disentangle the effects of time and social experience (O'Rourke and Mendelson 2013). For instance, in two-spotted gobies, the availability of mates declines dramatically over the course of the breeding season (Forsgren et al. 2004) and in accordance with that change, females become unselective with respect to male size at the end of the season (Borg et al. 2006). However, in addition to experiencing a change in OSR over the season, females are aging, which may also lead to less stringent mating requirements (Real 1990).

To predict how mate choice is likely to evolve in dynamic environments, it is not enough to simply document variation in mate choice that is associated with environmental parameters. It is also necessary to understand (1) the heterogeneity of the environment in which the behaviors of interest evolved, (2) the dependence of current behavioral responses on extrinsic and intrinsic factors, and (3) how these factors interact with one another (Tuomainen and Candolin 2011; Candolin and Wong 2012). Understanding the environmental heterogeneity in which current behaviors evolved will inform whether animals are likely to have reaction norms that will allow them to cope with new conditions. Here, we consider all three of these points, using both data collected from field surveys as well as from laboratory experiments where we manipulate both intrinsic and extrinsic factors simultaneously.

**Figure 1:**
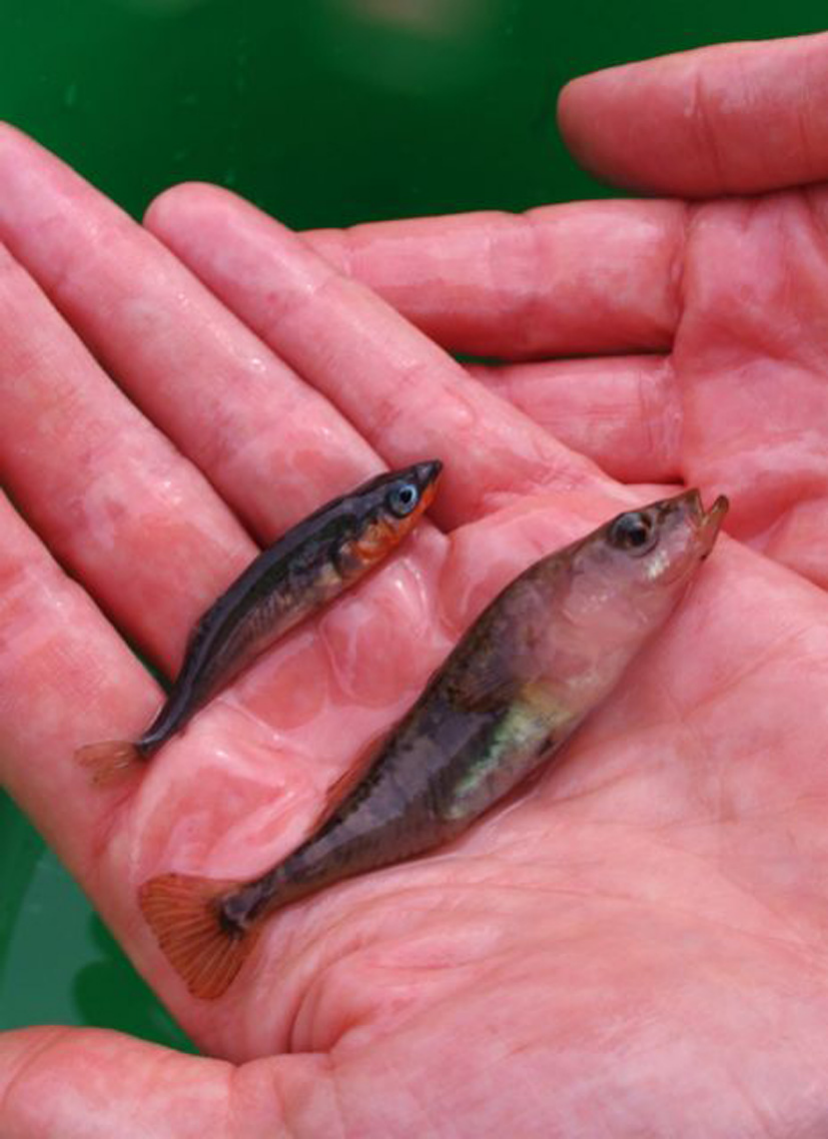
Limnetic (top) and benthic (bottom) male threespine stickleback from Paxton Lake, British Columbia.

Our study system is the limnetic–benthic species pair of threespine sticklebacks (*Gasterosteus* spp.) from Paxton Lake, British Columbia (Fig. 1). Limnetic and benthic sticklebacks are young species and appear to be capable of very rapid changes in mate choice (Milinski and Bakker 1992; Kozak et al. 2012), including occasional hybridization. In newly differentiated groups in which mate preferences contribute to reproductive isolation, knowledge of the environmental variation leading to changed mating decisions may be particularly important because species boundaries may still be fragile. For instance, in Enos Lake, a well established stickleback species pair collapsed into a hybrid swarm in fewer than 20 generations following the introduction of a nonnative crayfish that radically altered the lake macrophyte structure and thus the environment in which mate choice takes place (Gow et al. 2006; Taylor et al. 2006; Behm et al. 2010).

In this study, we first investigated temporal and spatial variation in mate availability in Paxton Lake, and then manipulated mate availability (sex ratio) in the lab, following individuals as they aged through an entire breeding season. If Paxton Lake sticklebacks regularly experience variation in mate availability within seasons and lifetimes, they may have evolved highly plastic preferences that facilitate changes in mate choice. In our field study, we sampled both limnetics and benthics because the number of males of each type (mate availability) may alter reproductive isolation between the two species via changes in female choice. In our lab manipulation, we worked with limnetic sticklebacks, which live for only 1 year (COSEWIC 2010). This means that age and time of season are synonymous in this system.

Our design allowed us to assess whether and how sexual signals, male–male competition, and female choice respond to changes in mate availability, and whether and how plasticity in mate choice depends on age. There have been numerous recent criticisms of the simplistic predictions of OSR theory (e.g., Klug et al. 2010; Weir et al. 2011), and age can impact mating decisions in a number of ways. We therefore present a number of alternative experimental outcomes. If the sexes respond to sex ratio but not age, according to classic OSR theory, male–male competition and sexual signaling should be at their highest and females most choosy when the sex ratio is male biased (Kvarnemo and Ahnesjo 1996). Alternatively, however, male–male competition and sexual signaling may be low at very male biased sex ratios, because it is no longer optimal to invest in these costly behaviors when females are rare and rivals more numerous (Weir et al. 2011). If the sexes respond to changes in life-history (or similarly, season), but not the social environment, life-history theory would predict that late in life when residual reproductive value is declining, males may increase competitive behavior and sexual signaling and females may become less choosy as a “last ditch effort” to achieve some reproductive success (Real 1990; Moore and Moore 2001). Alternatively, males may instead decrease investment in future (as opposed to current) reproduction late in life by decreasing signaling and competition and instead focusing on parental care, and females, once mated, may accept only very high-quality mates late in life (Jennions and Petrie 2000). Given these alternatives, a number of outcomes may result from interactions between experience with mates and age. One general prediction is that male competition will be highest and female mate choice most relaxed under male-biased conditions late in the breeding season. Manipulating the sex ratio and following competition and courtship as individuals age will allow us to tease apart the effects of social experience and life-history on mating decisions.

## Methods

### Spatial and temporal variation in demography in the field

Limnetic and benthic threespine sticklebacks were trapped in Paxton Lake (British Columbia, Canada) in 2007 to document spatial and temporal variation in mate availability in the field. Sampling was conducted at three time points during the breeding season, 4th to 5th April (early), 14th and 28th May (mid), and 15th to 17th June (late). Four sampling sites were chosen throughout the lake to represent different types of habitat that fish are likely to use for breeding. At each site, we placed 2–4 transects (transects were nested within sites). Most site and transect locations were consistently sampled throughout the breeding season, although the length of transects varied across sites and time points depending on visibility and topography, and some transects were not sampled at all three time points. To assess OSR, we set minnow traps at 2–4 m intervals along the length of each transect. We also estimated % vegetation cover and water depth in quadrats surrounding the traps, as these habitat variables are important ecological predictors of where the two species are likely to be found and to breed (Schluter 1993). We set 44 traps at the early time point, 47 traps at the mid-season time point, and 51 traps at the late time point. Traps were placed where males were nesting. Fish caught in traps were identified as reproductive or nonreproductive, benthic or limnetic, and if reproductive, male or female. Only reproductive fish were considered in our estimates of OSR. Males were identified by breeding coloration, and females by the presence of eggs. We cannot be sure that all males in breeding coloration were nest holders, but, all males expressing nuptial coloration are capable of mating and sneaking is common in sticklebacks (Wootton 1984). Thus, we believe that including all males that express nuptial coloration in our estimates of OSR is more accurate than only including the number of nest-holding males. In the context of this study, what's most important is assessing whether females experience variation in sex ratio over time or space.

### Manipulating mate availability

#### Maintenance of experimental fish

We collected wild reproductive limnetic sticklebacks using minnow traps in Paxton Lake at the beginning of the breeding season in 2011. Limnetics were chosen as our focal study species, in part, because they mature after 1 year and rarely live beyond a single breeding season (COSEWIC 2010). This is likely to make them particularly sensitive to within season variation in mate availability and means that as a single breeding season progresses, females approach the end of their reproductive lives. Fish were transported from British Columbia to Michigan State University where they were housed in 284-L tanks, each of which contained 16 fish in either a 1:3 or 3:1 ratio of males to females. Tanks also included inverted ½ flower pots and plastic plants for cover. Individuals were randomly assigned to treatments and replicate tanks, and treatment tanks were visually isolated from one another. We did not provide males in the 284-L treatment tanks with nest building materials, to avoid spawning events in the treatment tanks that would vary females' mating status, but individuals nevertheless readily exhibited both male–male competition and courtship behaviors as described below. We fed the fish bloodworms (*Chironomus* spp.) daily and maintained them under summer conditions with 14-h day lengths and a room temperature of approximately 18°C. All individuals were marked with colored elastomer to facilitate individual identification. To achieve a balanced design, we followed four focal females from each of the treatment tanks (randomly chosen among the 12 females in the female-biased tanks) throughout the breeding season to assess changes in mate choice using the courtship trials described below.

A number of individuals did not survive through the entire season. When an individual in a treatment tank died we replaced him/her with a previously unassigned individual to keep the density and sex ratio consistent throughout the season. We did not, however, collect mate choice data for females who were added to treatment tanks part way through the experiment.

#### Female experience with signaling and male competition

Within treatment tanks (described above) we monitored male sexual signals and competition throughout the season. Early, mid, and late in the breeding season (at approximately 4 week intervals beginning 4 weeks after the treatment tanks were established) we used an event recorder (Observer: Noldus Technologies, Wageningen, The Netherlands) to record male–male competition behaviors (charges, stalking, herding, displacing, and mouth wrestling) during 20-min trials for each treatment tank. We also recorded the behaviors bite and chase, which are common in both male competition and courtship, but do not report on these measures here because of difficulty distinguishing whether they were directed at males or females. During the same three time periods, we assessed the throat color index of all males in treatment tanks using a standardized color scoring method developed by our lab group (Boughman 2001, 2007; Lewandowski and Boughman 2008) that closely matches reflectance data (Albert et al. 2007; Boughman 2007). In this protocol, male red throat color area and intensity are measured on a scale of 0–5, where 0 indicates no color and 5 indicates maximum color or intensity. We sum these two scores to get a throat color index for each male that ranges from 0 to 10.

#### Female mate choice

Each time a focal female from the treatment tanks developed a new clutch of eggs, she was used in three no-choice courtship trials with novel nesting males who had “dull,” “medium,” and “bright” nuptial throat color (in increasing order). Females had between 0 and 3 clutches throughout the season. Between courtship trials females were given two hours to rest alone in 38-L or 110-L tanks. This period of time should be sufficient to eliminate the effects of sequential mate choice on mating decisions in sticklebacks (Milinski and Bakker 1992). We tested females with dull males first to reduce the possibility that responses to dull males were dependent on experience with nesting males encountered earlier in the day. Trials with dull males, who have nonpreferred sexual signals, were particularly important because if females relax their mate choices under female-biased sex ratios or late in the season, they may be more likely to accept less desirable mates.

Nesting males used in the courtship trials were housed individually in visually isolated 110-L tanks containing an inverted ½ flower pot for cover, a plastic plant, and pieces of aquatic plant material (*Chara* spp) for nest building. Each day, prior to courtship trials, we scored nuptial coloration of males with complete nests, categorizing them as dull, medium, or bright. Limnetic females strongly prefer the reddest males, and male color is positively correlated with physical condition (Wootton 1976; Milinski and Bakker 1990; Bakker 1993; Boughman 2001). The nesting males were novel to tested females, and females never saw the same nesting male twice. Nesting males were used in up to three different courtship trials with three different females. Immediately before courtship trials, we recorded the nesting male's nuptial throat color using the color scoring methods described above. The males we assigned to the dull, medium, and bright categories differed significantly in throat color index (dull = 1.27 ± 0.172; medium = 3.51 ± 0.177; bright = 5.72 ± 0.176; *F*_2,193_ = 162.95, *P* < 0.0001).

For each courtship trial, a gravid female was placed in an opaque holding container in a nesting male's tank, and after a 5-min acclimation period, she was released into the tank. Trials began when the male and female first interacted. We used an event recorder (Observer: Noldus Technologies) to record all courtship behaviors for the duration of each 20-min trial, or until the female entered the male's nest. Our measures of female choice included responsiveness (the number of times a female followed a male when he led her to the nest; a measure of motivation), and preference score, which measures how far courtship progressed (ranging from no response to attempted spawning: 0–4; Kozak and Boughman 2009; Kozak et al. 2009). A female who is not choosy would attempt to spawn with every male encountered and thus have a consistent preference score of 4. A reviewer suggested that we instead call this measure “acceptance score.” We prefer to maintain the terminology “preference score” because it is consistent with previous studies by our lab group and others (e.g., Kozak and Boughman 2009; Kozak et al. 2009).

### Statistical analysis

#### Mate availability in the field

Because individual trapping locations were repeatedly surveyed throughout the breeding season, we used a restricted maximum likelihood (REML) mixed model with random intercepts to estimate how mate availability (OSR) was influenced by fish type (benthic or limnetic), seasonality (start, mid, end), breeding site (a, b, c, d), and ecological variables (% vegetative cover and water depth) in Paxton Lake. Trap was a random effect in the field data model, which included fish type as a fixed categorical factor, time of season as a fixed categorical factor, site and transects nested within sites as fixed categorical factors, water depth (m) as a continuous factor, and % cover as a continuous factor. Again, the four sampling sites were chosen to represent different types of habitat the fish are likely to use for breeding. OSR (proportion male) was arcsine-square root transformed to improve normality (Wilson and Hardy 2002) and observations (OSR in each trap) were weighted by the number of fish caught in each trap because OSR estimates are so dependent on sample size (Wilson and Hardy 2002). Traps that were brought up empty were entered as missing data. In all of the models we report, nonsignificant interactions were removed from the final model as failing to do so causes spurious conclusions about main experimental effects (Enqvist 2005). All analyses were performed in JMP v. 9, which utilizes the Satterthwaite procedure to calculate degrees of freedom (Fai and Cornelius 1996) in mixed models.

#### Female experience with male signaling and competition

We assessed nuptial throat color of all males from 12 treatment tanks (six replicates of male-biased sex ratio and six replicates of female-biased sex ratio) at three time points (early, mid, and late in the season). Again, we used a REML mixed model to assess whether male throat color indices varied throughout the breeding season, under alternative sex ratios, or with their interaction. Home tank (replicate) was a random effect in the model. We assessed female experience with male competition in home tanks in a similar way, using identical model parameters to reveal whether and how the frequency of male–male competition interactions varied over time and with alternative sex ratios. The response variable was the sum of male–male competitive behaviors for each tank. This allowed us to assess male competition from the perspective of what females experience in their environments at alternative sex ratios.

#### Female mating decisions

Female sticklebacks' responses to dull, medium, and bright males in courtship trials were quantified in two ways: responsiveness and preference score. Individual females were repeatedly tested within a given day and across clutches, so again, we used REML mixed models to estimate how the fixed factors clutch number, sex ratio treatment, trial type (dull, medium, or bright), and composite throat color in home tank affected female choice. The composite throat color in a female's home tank was measured as the sum of the throat color indices of all males in a female's treatment. We included this measure in our models because red throat color is a predictor of female interest in males, is phenotypically plastic, and males' color indices changed over the course of our experiment (see Results). Female ID was a random effect in both models. We report variation in mate choice across clutches to assess changes in female mating behavior that occur as females age and the breeding season progresses. Clutch number is significantly positively correlated with time of season (date) (Logistic Regression *R*^2^ = 0.26, likelihood ratio *χ*^2^ with 2 df = 106.39, *P* < 0.0001). Females who were never led to the nest by courting males were entered as missing data for responsiveness.

## Results

### 

#### Mate availability in the field

**Figure 2:**
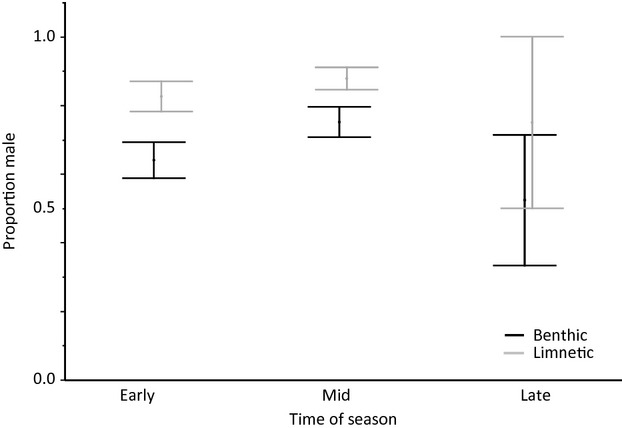
Operational sex ratio of limnetic (gray) and benthic (black) threespine stickleback from Paxton Lake, early, mid, and late in the breeding season. Means ± SE are illustrated.

Operational sex ratio in Paxton Lake varied spatially (across sampling sites) and with percent vegetation cover, species (limnetic or benthic), and the interaction between species and water depth (Table 1, Fig. 2). The OSR in Paxton differed across species and was more male biased for limnetics than benthics. The OSR was also more male biased in areas where there was more vegetative cover and shallow water (although water depth was only a significant predictor of OSR through an interaction with species; Table 1). This may reflect males preferentially establishing territories in areas that are good for nesting. The proportion of males varied across sites from 0.47 ± 0.0566 (SE) for benthics at site C to 0.926 ± 0.0554 (SE) for limnetics at site A. There was also a highly significant interaction between water depth and species on OSR (Table 1), indicating that limnetic and benthic males and females use habitat differently. For limnetics, OSR was more male biased in deeper water, and for benthics the OSR was more male biased in shallow water.

**Table 1:** REML mixed model of seasonal and spatial variation in Paxton Lake operational sex ratio

Source	df	*F* ratio	*P*
Time of Season (early, mid, late)	2121.6	0.845	0.432
**% Cover**	185.9	6.694	**0.0114**
Water Depth	1118.9	1.093	0.298
**Species (limnetic or benthic)**	1121.1	12.956	**0.0005**
**Site**	347.25	5.63	**0.0022**
Transects nested within sites	9121.6	1.519	0.1512
**Species × water depth**	1121	10.55	**0.0015**

Trap ID was a random effect in the model. Significant *P*-values are indicated in bold. REML, restricted maximum likelihood.

Across all four breeding sites surveyed in Paxton Lake, we found 446 limnetic and 382 benthic reproductively ready males early in the season, 360 limnetic and 430 benthic reproductively ready males mid-season, and 10 of each type late in the season. Variation in the abundance of limnetic and benthic males may lead to species–specific encounter rates that vary seasonally and spatially. To address this, we asked whether the ratio of reproductively ready limnetic to benthic males varied across breeding sites at two time points, early and mid-season. We did not assess spatial variation in species–specific encounter rates late in the season because there were too few reproductive males collected at each breeding site at that time point to do so meaningfully. Early in the season, the ratio of limnetic to benthic males differed significantly across sites (proportion limnetic males = 0.579 at site a, 0.885 at site b, and 3.073 at site c; *χ*^2^ with 2 df = 106.65, *P* = 0). No reproductive males were found at site d early in the season. Mid-season, reproductive males were found at all four sites and but the ratio of limnetic to benthic males did not differ significantly across sites (0.9803 at site a, 0.7903 at site b, 0.7 at site c, and 1.545 at site d; *χ*^2^ with 3 df = 5.939, *P* = 0.1146).

#### Male signaling and competition

**Figure 3:**
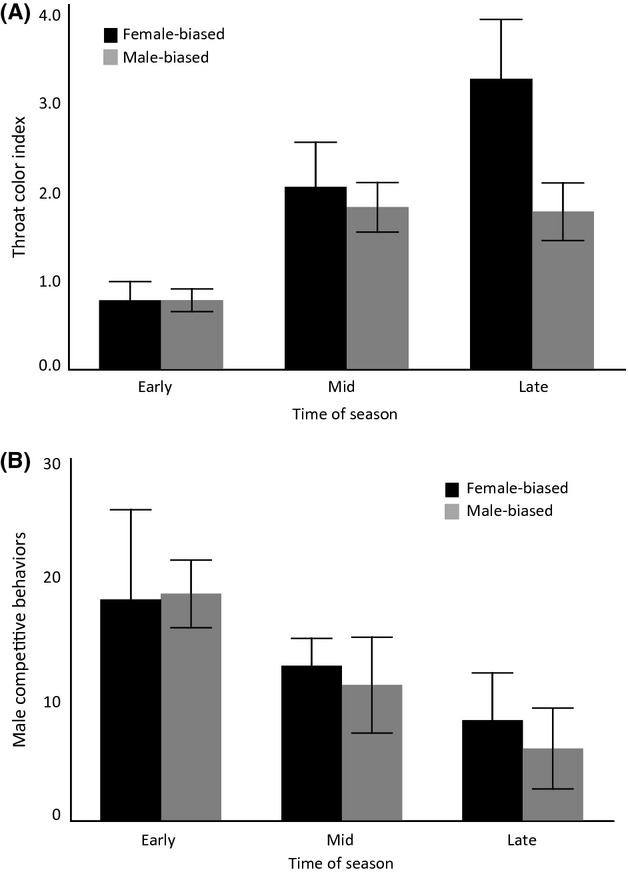
Female experience with nuptial throat coloration (0–10; A) and male–male competitive behavior (B) in female-biased and male-biased treatment tanks early, mid, and late in the breeding season. Means ± SE are illustrated.

Male throat color index increased over the course of the breeding season (*F*_2,207.5_ = 16.655, *P* = 0.0001), with males from both OSR treatments signaling most intensely at the end of the season (Fig. 3A). Viewed over the whole season, OSR itself was not a significant predictor of throat color index (*F*_1,16.2_ = 0.7788, *P* = 0.3904). However, there was a significant effect of the interaction between OSR and time of season on male throat coloration. Male throat color plateaued mid-season in male-biased tanks, but continued to increase late in the season in female-biased tanks (REML Mixed Model Parameter Estimate: Time(Late) × OSR Treatment *F*_1,207.7_ = 2.20, *P* = 0.029; *t* = 2.014, *P* = 0.0487; Fig. 2A).

Male competition behavior (charges + stalking + herding + displacing + mouth wrestling) was most intense early in the season, and declined as the season progressed (*F*_2,25.03_ = 3.43, *P* = 0.048; Fig. 3B). The number of male–male competition behaviors occurring in male-biased and female-biased tanks did not differ (*F*_1,25.33_ = 0.027, *P* = 0.871), nor did that depend on time of season (*F*_2,25.03_ = 0.0612, *P* = 0.941).

#### Female mating decisions

**Figure 4:**
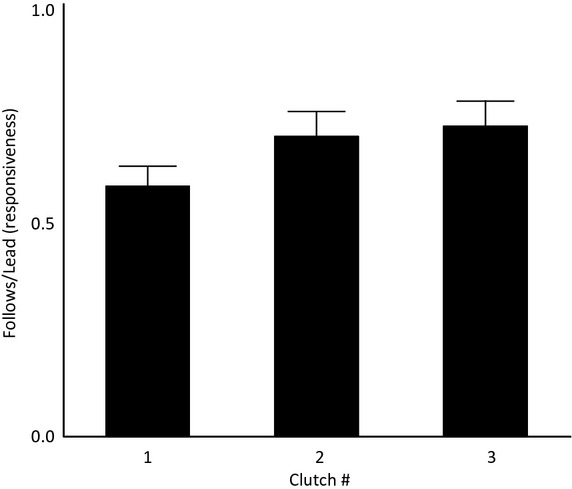
Responsiveness (follows/lead) of females as they aged (over three clutches). Females became more responsive as they aged, but sex ratio did not influence responsiveness. Means ± SE are illustrated.

Female responsiveness (follows per lead) increased as females aged and the breeding season progressed. This is evident from the significant relationship between clutch number and female responsiveness (*F*_2,147.6_ = 6.5407, *P* = 0.0019; Fig. 4). OSR treatment did not influence female responsiveness (male biased: 0.761 ± 0.073; female biased: 0.628 ± 0.095; *F*_2,38.72_ = 1.4532, *P* = 0.235). Females were equally responsive to males regardless of their color score (dull: 0.694 ± 0.064, medium: 0.721 ± 0.063, bright: 0.709 ± 0.064, respectively; *F*_2,124.7_ = 0.1023, *P* = 0.9028), but were more responsive when the composite color score they experienced in their OSR treatment tank was higher (*F*_1,105.5_ = 3.978, *P* = 0.0487).

**Figure 5:**
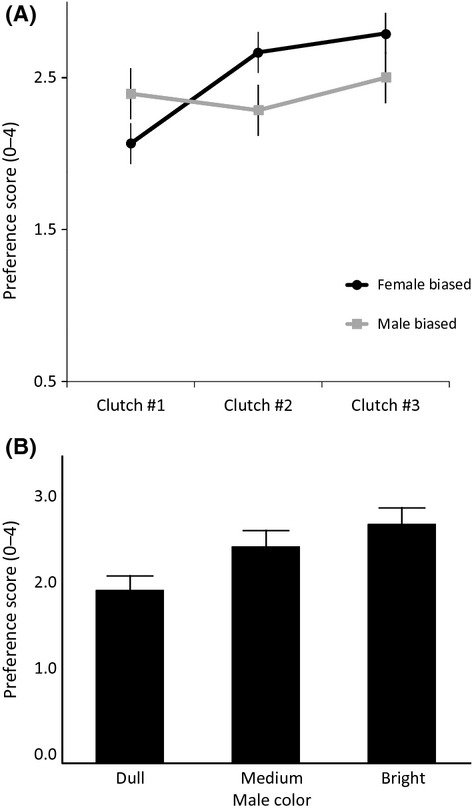
Preference score (0–4; A) of females from female-biased (black) and male-biased (gray) treatments as they aged (over clutches 1, 2, and 3). Preference score measures how far in the courtship sequence a given courting pair proceeded. Females from female-biased, but not male-biased treatments, increased their preference scores with all males, regardless of color, as they aged. Females maintained their preference for bright males over mid and dull ones, regardless of age and sex ratio (B). Means ± SE are illustrated.

Similarly to female responsiveness, female preference scores increased later in life – females proceeded further in the courtship sequence with their second and third clutches than their first (*F*_2,173.5_ = 3.348, *P* = 0.0374; Fig. 5A). Preference scores were also influenced by male coloration. Females proceeded further in the courtship sequence with males exhibiting greater red nuptial coloration (*F*_2,147.6_ = 6.203, *P* = 0.0026), corroborating previous work showing that they prefer males with more red color (Fig. 5B). Further, contrary to expectations, we found no main effect of OSR on female preference score (*F*_1,39.3_ = 2.276, *P* = 0.1394), but OSR did influence preference score via an interaction with age (OSR × Clutch Number, *F*_2,167_ = 3.07, *P* = 0.048; Fig. 4A). Females from female-biased tanks had higher preference scores with later clutches, while females from male-biased tanks did not change their preference scores as they aged. The sum composite color score that females experienced in their home treatment tanks did not impact how far into the courtship sequence females proceeded during courtship trials (*F*_1,97.94_ = 2.2519, *P* = 0.1367). It is worth noting that male coloration was not involved in significant interactions with either clutch number or OSR treatment in models of responsiveness or preference score, indicating that preference functions did not vary with age or sex ratio.

## Discussion

Much of the observed variation in mate choice may be due to adaptive plasticity that allows females to alter mating decisions depending on both their attributes and their circumstances. Here, we asked how variation in abundance of potential mates impacts sexual signaling, male competition, and mate choice, and importantly, whether and how the intrinsic factor age alters those behavioral responses. In short, we find that both male sexual signaling and female mating decisions (choosiness) are influenced by the interaction of age and mate availability, but male competition is not.

Our observations of male signaling and competition behavior provide information about the social experiences that females have under conditions of high and low mate availability. Consistent with life-history theory (Real 1990) and previous work in sticklebacks (Candolin 1999, 2000), we found that male nuptial coloration was plastic (Barber et al. 2000), with males in both sex ratio treatments developing more intense red nuptial throat coloration late in the season. This tendency was greater in males from female-biased tanks than male-biased tanks (Fig. 3A). The plasticity in signaling we observed does not appear to be explained by the intensity of competition early, mid, and late in the season (Fig. 3B). Instead, we suggest that at the end of the breeding season, the cost of signaling, in terms of lost longevity, is low (Candolin 1999). In response to changes in the cost of signaling with time, males increase investment in reproduction by signaling maximally when females are abundant.

Why don't males from male-biased tanks signal maximally late in life? We suggest three explanations. First, this result supports the hypothesis that when females are rare, it may not be optimal for males to invest in such costly signals, at least not late in life. Second, the presence of a greater number of competitors in male-biased tanks may maintain honesty in signals (Candolin 2000). If all males in female-biased tanks can establish territories, but at male-biased sex ratios, territories are limiting, red may be a badge of status, with males of low status decreasing red to escape fights. Candolin (2000) found a similar effect in sticklebacks allowed to court either alone or with competitors. When competitors were present, males decreased their nuptial coloration, honestly indicating their parental ability, rather than signaling maximally. This may also explain why we don't find differences in the number of male–male competitive interactions in male-biased and female-biased tanks, if lower ranking males in male-biased tanks avoid direct competition by “dialing down” their signals. Finally, we can hypothesize a proximate explanation. If red coloration is responsive to the frequency of encounters with females, we would find males from female-biased treatments to be more red.

We also observed an effect of “habituation” on male–male competition, whereby males in both OSR treatments engaged in male–male competition vigorously early in the season, but did not maintain these levels of interaction throughout the season (Fig. 3B). Male competition, then, responds to changes in life-history as well, but in the opposite direction from sexual signaling. This could reflect a trade-off between sexual signaling and male competition, such that males invest more in courtship and less in competition as they age. Alternatively, this may be interpreted as a dear enemy effect; once territories have been established, fewer direct male competition interactions are needed to maintain existing relationships (reviewed in Temeles 1994). A similar decrease in aggression toward neighbors over time has been found in many taxa (Temeles 1994) including sticklebacks (Rowland 1988).

Simultaneous with the time of season when males invested most in signaling, aging females were most responsive to courting males (Fig. 4), and proceeded further in the courtship sequence when they encountered nesting males (Fig. 5A). This effect is consistent with work in other systems (e.g., cockroaches (Moore and Moore 2001). The pattern, however, is driven largely by individuals from female-biased tanks, who proceed further in the courtship sequence late in the season than do individuals from male-biased tanks. In other words, changes in female choosiness that are associated with aging also appear to depend on social experience with potential mates. Are females “primed” to accept males more readily at the end of the season because males are signaling maximally? Do their decisions simply reflect an increased interest in mating as their reproductive lives come to an end? Females appear to maintain their preferences for the reddest males throughout the breeding season (preferring medium and bright males over dull males at all time points; Fig. 5B), but proceed further in the courtship sequence with all males (including less-preferred, lower quality dull males) late in the season, especially when they've experienced female-biased sex ratios. In other words, we found no evidence of a significant interaction between male color (dull, medium, and bright) and time of season on preference scores or responsiveness. Females are more responsive and less choosy across the board when they age. Although, on average, male throat color index was greatest late in the season (Fig. 3A), we found no effect of the sum composite color that females experienced in their treatment tanks on preference score, suggesting that females are not “primed” to accept males more readily at the end of the season simply because males are more red then.

Our field survey of mate availability revealed that Paxton Lake sticklebacks are likely to experience variation in OSR within breeding seasons as they move through the lake in search of nesting males in likely breeding locations. This supports work in other systems, where OSR has been found to vary within individuals' lifetimes and single breeding seasons (e.g., pipefish [Vincent et al. 1994], two-spotted gobies [Forsgren et al. 2004], and fantail darters [O'Rourke and Mendelson 2013]). Previous reports in which Paxton Lake sticklebacks were designated as endangered assumed an equal sex ratio (COSEWIC 2010). Instead, we find that OSR varies from approximately 1:1 to >90% male across sites and depending on species. This spatial variation in OSR appears to be related to the quality of nesting habitat. OSRs were more male biased at sites with a high percentage of vegetative cover, a characteristic of good nesting habitat in other populations (Candolin and Voigt 1998). Taken together, our lab and field results suggest that changes in “desirable” mating habitat may have important consequences for female mating decisions which could then affect the maintenance of reproductive isolation: females experience variation in mate availability across sites within breeding seasons, and the interaction of mate availability and age leads females to make more relaxed mating decisions.

Hybridization between closely related species is becoming more common, but the relationship between mate choice and hybridization is not well understood (Willis et al. 2011). It remains to be seen, for instance, whether the types of variation in mating decisions observed here extend to interactions with heterospecifics. Future work is planned to address this question more fully, and we limit our discussion here to some related observations from this study. The flexibility built into mate choice to deal with temporal and spatial variation in environments may lead to increased hybridization when preferred mates are rare or hard to find (e.g., Seehausen et al. 2008). In our field study, we detected spatial variation in the species–specific encounter rate (estimated by the ratio of reproductively ready limnetic to benthic males caught in our traps) early in the breeding season, but not mid-season. Our lab study of female choice in the limnetic species, however, shows that females do not relax their mating decisions until later in life. Early in the season, when the availability of conspecific mates varies spatially, females are at their most discriminating, so experience with heterospecifics at this point in life may have little bearing on the maintenance of reproductive isolation. If conspecific mate availability is limited later in the season, however, when females are older, hybridization rates between limnetics and benthics may increase. Flexibility in mating behavior can be adaptive in the sense that it facilitates gaining some reproductive success as opposed to none, but when ecosystems are disturbed, what once was adaptive plasticity could instead lead to biodiversity loss through hybridization (Pfennig 2007).

What does the flexibility in mating decisions that we've observed tell us about how we should expect mate choice to evolve in response to changing sexual selection when environments and population ecology change? The sexual selection literature overwhelmingly assumes that females do not have a hard time finding mates (Bateman 1948; Trivers 1972; Andersson 1994), although recent work has highlighted how dynamic mate availability and mating decisions can be (e.g., Forsgren et al. 2004; Borg et al. 2006; Myhre et al. 2012). Anthropogenic disturbances will alter the selection pressures that mold mate choice, and the nature of current behavioral flexibility will determine how individuals respond to new environmental perturbations. For instance, flexibility in mate choice in response to experience with mates has been suggested to be important for the process of colonization where appropriate or preferred mates may be hard to come by, by increasing the invasive capacity of populations through the reduction of Allee effects (Vargas-Salinas 2006; Fowler-Finn and Rodriguez 2011). We suggest that the same mechanisms may facilitate the maintenance of populations that are experiencing range expansion or contraction, invading new habitats, undergoing harvesting or high rates of predation, and habitat fragmentation. This study adds to our growing understanding of flexible mate choice by placing that flexibility in a life-history context. We show that female mating decisions can depend on the interaction of experience with mates and age. Understanding current environmental heterogeneity and patterns of flexibility in mating behavior will allow us to better predict how populations are likely to respond to changing sexual selection when we alter the environment.
